# Pseudofinder: Detection of Pseudogenes in Prokaryotic Genomes

**DOI:** 10.1093/molbev/msac153

**Published:** 2022-07-08

**Authors:** Mitchell J Syberg-Olsen, Arkadiy I Garber, Patrick J Keeling, John P McCutcheon, Filip Husnik

**Affiliations:** Department of Botany, University of British Columbia, Vancouver, BC, Canada; Division of Biological Sciences, University of Montana, Missoula, MT, USA; Department of Botany, University of British Columbia, Vancouver, BC, Canada; Division of Biological Sciences, University of Montana, Missoula, MT, USA; Howard Hughes Medical Institute, 4000 Jones Bridge Road, Chevy Chase, MD, USA; Department of Botany, University of British Columbia, Vancouver, BC, Canada; Okinawa Institute of Science and Technology, Okinawa, Japan

**Keywords:** *dN/dS*, bacteria, archaea, genome, pseudogene, annotation, prediction

## Abstract

Prokaryotic genomes are usually densely packed with intact and functional genes. However, in certain contexts, such as after recent ecological shifts or extreme population bottlenecks, broken and nonfunctional gene fragments can quickly accumulate and form a substantial fraction of the genome. Identification of these broken genes, called pseudogenes, is a critical step for understanding the evolutionary forces acting upon, and the functional potential encoded within, prokaryotic genomes. Here, we present Pseudofinder, an open-source software dedicated to pseudogene identification and analysis in bacterial and archaeal genomes. We demonstrate that Pseudofinder’s multi-pronged, reference-based approach can detect a wide variety of pseudogenes, including those that are highly degraded and typically missed by gene-calling pipelines, as well newly formed pseudogenes containing only one or a few inactivating mutations. Additionally, Pseudofinder can detect genes that lack inactivating substitutions but experiencing relaxed selection. Implementation of Pseudofinder in annotation pipelines will allow more precise estimations of the functional potential of sequenced microbes, while also generating new hypotheses related to the evolutionary dynamics of bacterial and archaeal genomes.

## Background

Pseudogenes are remnants of genes that have fixed inactivating nucleotide substitutions or insertions/deletions (indels) relative to their ancestral coding sequences (Lerat and Ochman 2005; Ochman and Davalos 2006). In eukaryotic genomes, pseudogenes frequently arise from relaxed selection on one copy of a gene resulting from gene (or whole genome) duplications, and much effort has gone toward specific studies, tools, and databases to identify them (Karro et al. 2007; Pink et al. 2011). In contrast, genomes of Bacteria and Archaea are usually gene dense and encode very few pseudogenes (Kuo et al. 2009; Kuo and Ochman 2010; Goodhead and Darby 2015). However, pseudogenes do exist in prokaryotic genomes (Liu et al. 2004; Lerat and Ochman 2005), most abundantly in species where large numbers of genes have become unnecessary through rapid and sustained changes in ecological context (Ochman and Davalos 2006). Classic examples include intracellular bacterial endosymbionts or pathogens, where, in extreme cases, pseudogenes can outnumber functional genes in a genome (Toh et al. 2006; Burke and Moran 2011; McCutcheon and Moran 2011; Singh and Cole 2011; Clayton et al. 2012; Oakeson et al. 2014; Danneels et al. 2018).

Identification of pseudogenes is critical for understanding the physiology, metabolism, and evolutionary adaptations of pathogens and symbionts. It is also an underappreciated step in the annotation of free-living bacterial genomes. Precise pseudogene annotation is important for bacterial and archaeal phylogenomics, as the inclusion of pseudogenes in phylogenetic trees may lead to artifacts such as overestimation of branch lengths. Despite the importance of pseudogene identification, it is still common for pseudogenes to be annotated manually based on arbitrary criteria, using custom unpublished scripts, or by relying on automatic annotation tools, such as the National Center for Biotechnology Information (NCBI) prokaryotic genome annotation pipeline (PGAP, Tatusova et al. 2016) or DFAST (Tanizawa et al. 2018). These tools, designed primarily for functional gene annotation, are not ideal for pseudogene prediction because they lack standardization and do not allow for species-specific adjustments.

A handful of tools for pseudogene prediction have been developed over the last two decades. While the majority of these tools are designed and optimized for mammalian genomes (van Baren and Brent 2006; Zhang et al. 2006; Ortutay and Vihinen 2008; Campbell et al. 2014; Alves et al. 2020), several pseudogene-relevant tools have been designed with prokaryotic genomes in mind (Psi-Phi: Lerat and Ochman 2005; PEPPAN: Zhou et al. 2020; Junker: Sridhar et al. 2011; SearchDOGS: Óhéigeartaigh et al. 2014; Beacon: Kalkatawi et al. 2015). However, these implementations are either not open-source, specifically designed for use in pangenomic analysis, or not specifically designed for pseudogene prediction and analysis (e.g., they do not allow changing parameters).

Here, we present Pseudofinder, an open-source and highly customizable program that differentiates candidate pseudogenes from intact genes in prokaryotic genomes. Pseudogene identification is guided by a reference-based approach where a genome-of-interest is annotated by comparison to a user-supplied protein sequence database (e.g., RefSeq, Pruitt et al. 2007) and/or a closely related reference genome. Using the reference database of proteins, Pseudofinder makes evidence-based annotations of truncated, fragmented, and highly degraded genes. When a reference genome of suitable evolutionary distance is available, Pseudofinder has the capacity to detect cryptic pseudogenes (or genes that have not accumulated inactivating substitutions but that may be experiencing relaxed selection), and reports on the type and quantity of any inactivating mutations that it detects (e.g., nonsense substitutions, frameshift-inducing indels, etc.).

## New Approaches

Pseudofinder is a new open-source software for generalized pseudogene prediction and evolutionary inference in prokaryotic genomes. Pseudofinder uses a reference-based approach to detect a wide variety of pseudogene-inducing substitutions and gene structures, giving users many customizable options for defining pseudogenes. This software can use comparisons to large protein databases and/or closely related genomes to interrogate pseudogenes and selective pressures at the detailed gene-by-gene level. Pseudofinder represents a new approach to pseudogene detection in prokaryotes because it is completely open-source, allows for user-defined customization, provides visualizations to allow users to explore their data with respect to pseudogenes, and generates evolutionary inferences based on a closely related reference genome (if available).

## Results and Discussion

Because finding pseudogenes in a genome is nontrivial, it is difficult to compare different approaches to the problem because the true set of pseudogenes for a given genome is unknown. To first demonstrate Pseudofinder’s capacity to accurately and precisely predict potential pseudogenes based on a defined set of criteria, we used the *Break* module to randomly generate pseudogenes within the genome of *Shigella flexneri* (supplementary fig. S1*A*, Supplementary Material online). Pseudogene creation using the *Break* module was performed ten times, each time increasing the number of pseudogene-forming substitutions introduced into the genome (supplementary fig. S1*B*, Supplementary Material online). Each pseudogene was generated randomly, so we could not predict exactly how each gene may or may not have been affected. However, the final set of pseudogenes produced with each simulation was determined by the same parameters that would then be used to detect those pseudogenes. We find that Pseudofinder detected nearly every *in silico*-generated pseudogene (supplementary fig. S1*C*, Supplementary Material online). For the purposes of this benchmarking, natural pseudogenes present on *Shigella’s* genome before *in silico* mutagenesis were ignored.

Next, we tested Pseudofinder with two bacterial genomes: 1) *Ca.* Sodalis pierantonius str. SOPE (Oakeson et al. 2014, hereafter *Sodalis*), a host-beneficial intracellular symbiont known to have many pseudogenes and 2) *Shewanella* sp. ZOR0012 (Lebov et al. 2020, hereafter, *Shewanella*), a strain closely related to *S. oneidensis* MR-1. *Shewanella sp.* ZOR0012 is not known to encode many pseudogenes, but due to its recent ecological niche change to the zebrafish intestinal tract, it may be experiencing a shift in selective pressures, particularly in relation to other metal-reducing *Shewanella* spp. (e.g., *Shewanella* MR-1, its closest known relative). We compared pseudogene predictions from Pseudofinder to those derived from two annotation pipelines that include pseudogene prediction as part of their workflow: PGAP (Tatusova et al. 2016) and DFAST (Tanizawa et al. 2018). Because DFAST provides the option of annotation using two different gene-prediction software packages (Prodigal, Hyatt et al. 2010 and MetaGeneAnnotator, Noguchi et al. 2008), which may differ in the genes predicted, we ran DFAST using both gene-calling methods. Additionally, it is worth noting that PGAP uses GeneMark for gene prediction, which may also result in differences in gene predictions. Considering these potential differences, we included in our benchmarking only those genes that were predicted by all three gene-calling pipelines. Consequently, we excluded pseudogene candidates identified by Pseudofinder in intergenic regions (i.e., regions between genes where no open reading frame is detected). We used *Shewanella* MR-1 and *Sodalis praecaptivus* HS as reference genomes for pseudogene analysis.

**Fig. 1.**
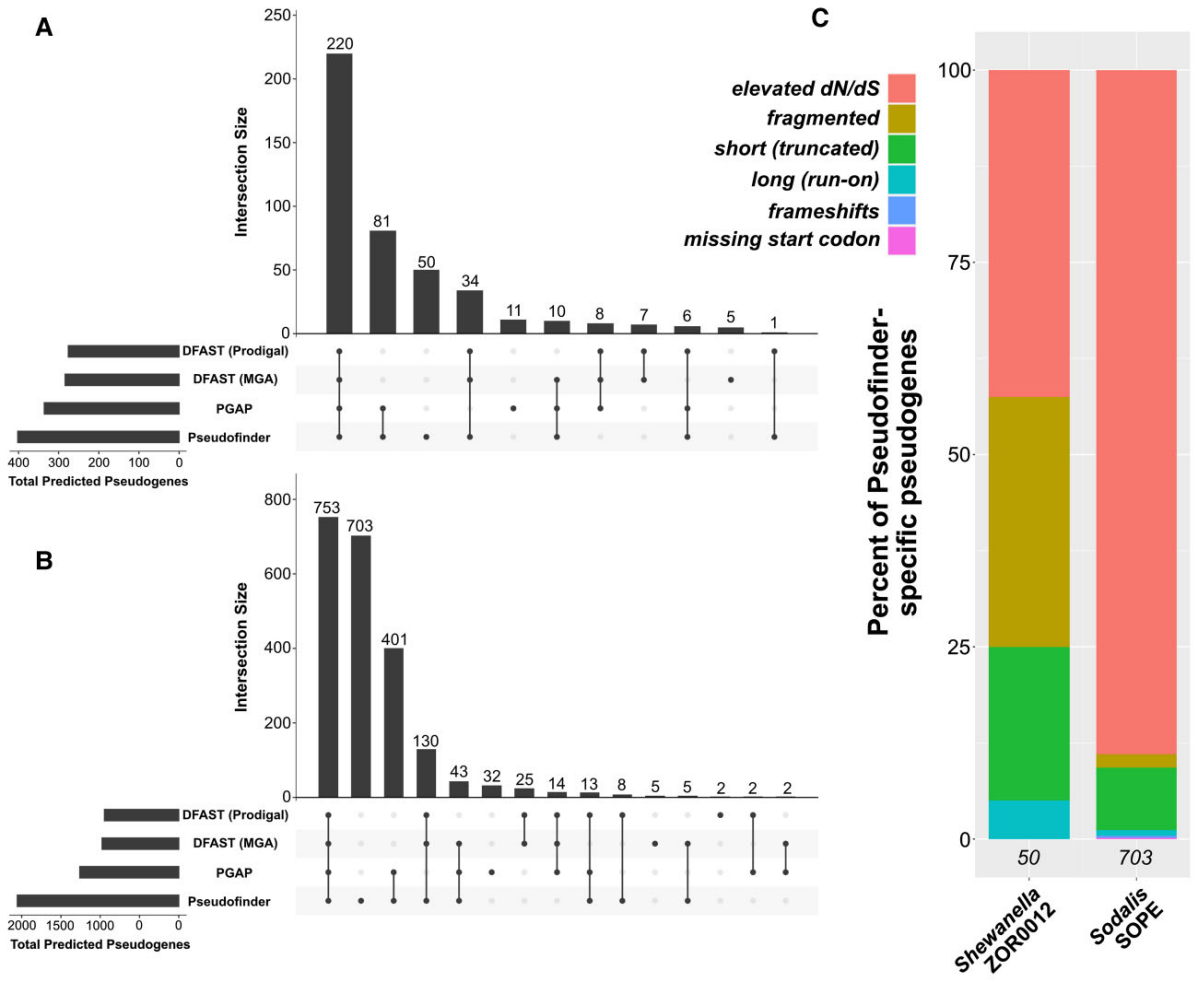
Summary of benchmarking results, comparing pseudogene predictions by Pseudofinder to those of two other softwares: PGAP and DFAST (run with two different gene-callers). (*A* and *B*) ‘Upset’ plots (Conway et al. 2017), showing the overlap and differences between the three pipelines in pseudogenes predicted from *Shewanella* (*A*) and *Sodalis* (*B*). Each bar in the barplot represents the total number of pseudogenes that overlap between the pipelines denoted with dots below. (*C*) Barplots showing the types of pseudogenes that were predicted only by Pseudofinder in *Shewanella* and *Sodalis* (i.e., Pseudofinder-specific pseudogenes). Italicized numbers at the bottom of each bar indicate the number of Pseudofinder-specific pseudogenes predicted in each genome.

In both genomes, Pseudofinder predicted the greatest number of pseudogenes compared with PGAP and DFAST. Of all Pseudofinder-identified pseudogenes, 87.6% (*Shewanella*) and 65.8% (*Sodalis*) were also flagged by at least one other annotation software (fig. 1*A*). The remaining 12.4% and 34.2% can be considered Pseudofinder-specific pseudogene candidates; these were flagged by Pseudofinder for a range of different reasons, the most common being elevated *dN/dS* (fig. 1*B*), a metric not used by DFAST or PGAP. *Sodalis* in particular encodes a large number of genes with elevated *dN/dS* (consistent with findings in Oakeson et al. 2014), which make up ∼90% of the Pseudofinder-specific pseudogene candidates in that genome. In this context, we interpret elevated *dN/dS* in a gene as relaxed selection. Because elevated *dN/dS* values are only suggestive of relaxed selection, we note that they should be interpreted cautiously and only as a hypothesis-generating exercise.

Genes considerably shorter than their top homologs from the reference database were also relatively common among the Pseudofinder-specific pseudogenes. Additionally, Pseudofinder identified gene remnants in genomic regions where no open reading frame was predicted (i.e., intergenic regions): 238 in *Shewanella* and 305 in *Sodalis*, but these counts are not included in the numbers presented in figure 1. Pseudogenes predicted by either or both PGAP or DFAST, but missed by Pseudofinder, represent 7.2% (*Shewanella*) and 3.8% (*Sodalis*) of the total predicted pseudogenes from each genome. PGAP- and DFAST-identified pseudogenes that were missed by Pseudofinder were manually inspected in reference to their top BLAST hits: many of these genes appear only marginally shorter than their top homologs (not enough to surpass the 75% length cutoff that we set for the Pseudofinder runs). This result emphasizes that arbitrary length cutoffs used for pseudogene identification should be interpreted with caution and, similar to *dN/dS* estimations, should be considered carefully by an expert user before reporting pseudogenes for a genome. Additionally, some of these genes did not recruit enough homologs from the reference database to be evaluated as pseudogenes by Pseudofinder. Importantly, both of these criteria can be adjusted by the user in Pseudofinder.

## Conclusions

We conclude that Pseudofinder accurately predicts pseudogenes based on user-specified criteria. We also find that Pseudofinder is more sensitive toward pseudogene identification than DFAST and PGAP. This sensitivity is primarily due to Pseudofinder including more metrics for pseudogenization (e.g., *dN/dS*); this is particularly apparent in the case of *Sodalis* str. SOPE, whose genome encodes many genes that appear to be under relaxed selection. Nonetheless, the differences identified here between pseudogene prediction by PGAP, DFAST, and Pseudofinder, demonstrate that identification of pseudogenes remains complicated and is best supplemented by manual inspection and curation. Pseudofinder offers a standardized pipeline and convenient package where users can easily tailor parameters relevant to the biological system at hand, visualize the results, and carry out *in silico* pseudogene simulations.

## Materials and Methods

### Software Description

Pseudofinder is implemented in Python 3. It has five built-in commands, or modules: *Annotate*, *Reannotate*, *Sleuth*, *Visualize*, and *Break*. The *Annotate* command performs the initial pseudogene analysis using a comprehensive database of proteins, such as RefSeq or NR (nonredundant database of proteins), available from NCBI. *Reannotate* is similar to *Annotate* but allows the user to bypass the most time-intensive steps of the pipeline and generate a new set of pseudogene predictions using different parameters. If a closely related reference genome is available, Pseudofinder uses the *Sleuth* module for reference-guided annotation, which can detect relaxed selection (via *dN/dS*), as well as the type and quantity of gene-disrupting mutations in each gene (e.g., frameshift-causing indels, loss of start/stop codons, nonsense substitutions, etc.). *Visualize* generates summary plots to assist the user in optimizing parameters for pseudogene identification. *Break* allows users to simulate pseudogenization of a genome *in silico*, randomly generating a set of pseudogenes along with a summary file that lists all created mutations and pseudogenes.

**Fig. 2.**
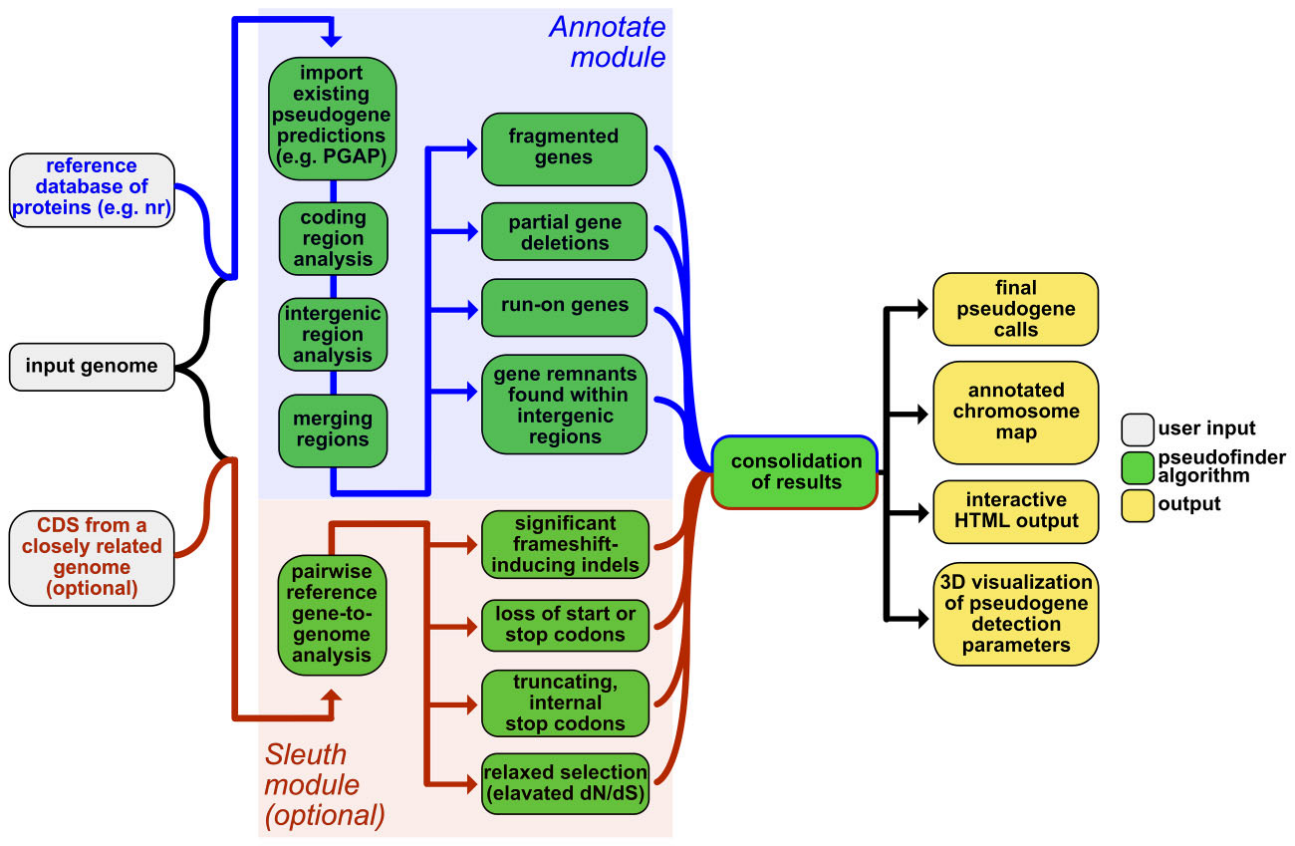
Pseudofinder workflow: the main *Annotate* branch is shown in the top part of the workflow, where predicted coding and intergenic regions are compared against proteins from a reference database, allowing the software to identify truncated and run-on ORFs, fragmented genes, and highly degraded gene remnants that lack identifiable gene features. The *Sleuth* branch is shown in the bottom part of the workflow, where genes from a closely related reference genome are compared against the genome-of-interest to identify gene inactivations at a finer scale; these inactivations, or gene breakages, can include significant frameshift-inducing indels (i.e., indels that results in substantial changes to the protein sequence), nonsense substitutions, loss of start and stop codons, and relaxed selection (elevated *dN/dS*, measured using PAML, Yang 2007). Information obtained from these two branches are then consolidated and provided to the user in the form of GFF and FASTA files for downstream processing. Pseudofinder also provides multiple ways for users to visualize the results, including a PDF-formatted genome diagram/map, as well as an HTML-formatted files for interactive exploration of pseudogene predictions.

- **Annotate:** This module represents Pseudofinder’s core pipeline. It accepts prokaryotic genomes in GenBank format (NCBI compliant, with both gene and CDS features) and a protein sequence database as input, along with many optional parameters. Additionally, users have the option of providing a single reference genome closely related to the query genome, in which case, the *Sleuth* module is invoked. The overall pipeline is outlined in figure 2. First, the input genome is split into coding regions and intergenic regions. Coding regions are predefined in the input annotation, and intergenic regions are defined as the regions between the predicted coding regions. For each coding region, homologs from the reference database are collected using BLASTP (Camacho et al. 2009) or DIAMOND (Buchfink et al. 2015). Truncated coding regions are identified by comparing gene and alignment lengths to the average lengths of top homologs identified from the reference database. Because genes naturally vary in size, in addition to an arbitrary length cutoff, Pseudofinder can consider the mean and standard deviation of the top DIAMOND/BLAST hits to each queried gene. Fragmented genes are identified as adjacently encoded gene fragments that share a single ancestral gene and, consequently, recruit the same homologs from the reference protein database (supplementary fig. S2, Supplementary Material online). For each intergenic region, BLASTX is used to check for significant amino acid sequence similarity in all six reading frames. This process recovers highly degraded pseudogenes that have been missed by gene-prediction software and can identify regions of pseudogenes upstream or downstream of predicted, truncated gene regions.
- **Sleuth:** While Sleuth is invoked when a reference genome is provided to *Annotate*, *Sleuth* is also a standalone module that accepts as input a prokaryotic genome and a reference genome’s CDS, and performs a pairwise analysis. First, CDS from the reference genome are queried against the genome-of-interest. Homologous regions are then realigned using Muscle (Edgar 2004), and the resulting alignments are processed with respect to indels, nonsense substitutions, frameshift-induced early stop codons, loss of start or stop codons, and *dN/dS*. The use of *dN*/*dS* should be restricted to genomes within a reasonable evolutionary distance (e.g., no more distantly related than at the genus level), and specific genes within a certain evolutionary divergence (e.g., *dS* > 0.01 and *dS* < 3, which are both set as defaults within the software). Moreover, the inference of relaxed selection from *dN/dS* values must be done in the context of the rest of the genome (Rocha et al. 2006). *Sleuth* also estimates the degree to which frameshift-inducing indels impact the resulting protein sequence: for example, frameshift-causing indels are considered deleterious when they significantly impact the amino acid sequence of the gene product. By comparing the Muscle-based nucleotide alignment to the protein-dependent codon alignment generated with pal2nal (Suyama et al. 2006), the *Sleuth* module measures the impact that frameshift-inducing indels have on the overall protein sequence and uses this information to predict pseudogenes (supplementary fig. S3, Supplementary Material online).
- **Visualize:** Annotating pseudogenes requires that we define biologically arbitrary cutoffs. For example, Pseudofinder has many parameters that can be tuned by the user, which have the potential to significantly impact pseudogene predictions. These parameters are arbitrary because genes naturally vary in size, the number of domains, the amount of frameshift-inducing indels they can tolerate, and their substitutions rates. In other words, a one-size-fits-all definition for pseudogenes is not appropriate. We urge users to test multiple settings and visualize each set of results using Pseudofinder’s *Visualize* module (in particular the *dN/dS* and length cutoff compared with reference sequences). This built-in visualization function helps to inform users how their results change as they modify various cutoffs. With a single command, a 3D plot will be generated using Plotly (Plotly Technologies Inc. 2015) to display the number of pseudogenes flagged (*z*-axis) with any combination of length and similarity parameters.
- **Break:** This module allows users to simulate pseudogenization in input genomes. *Break* will randomly generate a set of pseudogenes, given an input genome and a user-specified level of decay (1–10, 1 being the lowest and 10 being the highest level of decay) (supplementary fig. S1*B*, Supplementary Material online). Users must provide genome contig(s) or scaffold(s) as well as a corresponding GFF file. *Break* then randomly selects a number of genes (the number depends on the set level of decay) and performs one of five types of mutations (supplementary fig. S1*A*, Supplementary Material online). The specific details of how these mutations are chosen and then tracked are explained in Pseudofinder’s wiki: https://github.com/filip-husnik/pseudofinder/wiki.

## Supplementary Material

msac153_Supplementary_Data

## Data Availability

Pseudofinder is implemented in Python v3 and is freely available from https://github.com/filip-husnik/pseudofinder under the GNU General Public License v3.0.
