# Supplementary material for: Pseudofinder: Detection of Pseudogenes in Prokaryotic Genomes
Source: Mol Biol Evol. 2022 Jul 8;39(7):msac153. doi: 10.1093/molbev/msac153 (PMC9336565; doi:10.1093/molbev/msac153)
Supplement: msac153_Supplementary_Data [file msac153_supplementary_data.docx]

**
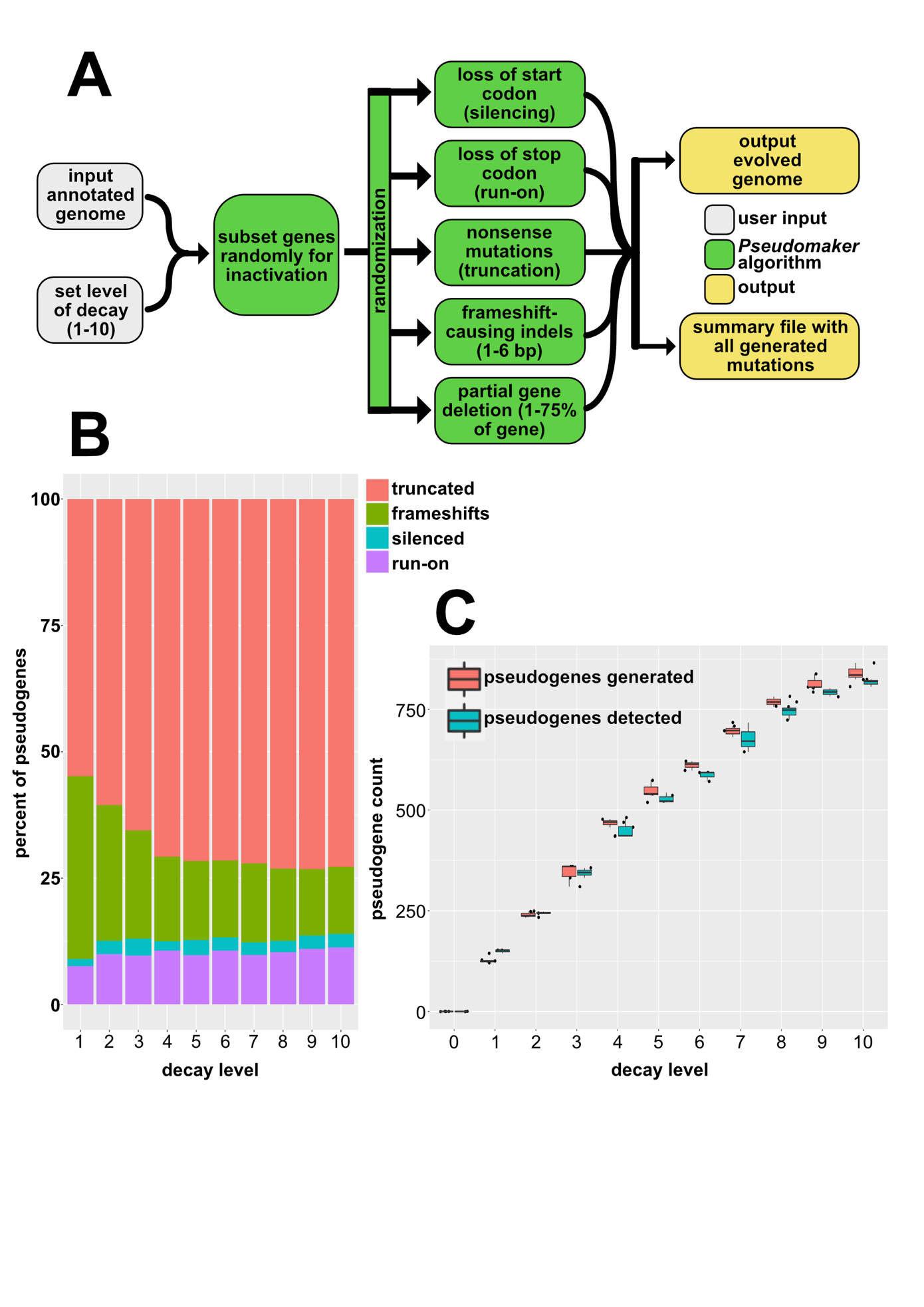
**

**Supplemental Figure S1** | A) Workflow of Pseudofinder’s *Break* module. The level of decay controls how many of the input genes are randomly chosen for one of the five types of mutations shown in green (e.g. silencing, truncation). The input genome must be provided as contigs in FASTA format, along with a GFF file describing annotations. The output (shown in yellow) features contigs with the randomized mutations in FASTA format, along with a summary file that lists the genes randomly chosen mutation, the types of mutations, and the resulting effects of those mutations (e.g. truncation, partial gene deletion, irreparable frameshifts, etc.) B) Barplot showing the types of pseudogenes formed, as a percent of total pseudogenes formed, after *in silico* mutations were introduced by the *Break* module. C) The total counts of pseudogenes created using the *Break* module, and then subsequently detected using Pseudofinder’s *Annotate* and *Sleuth* modules.

**
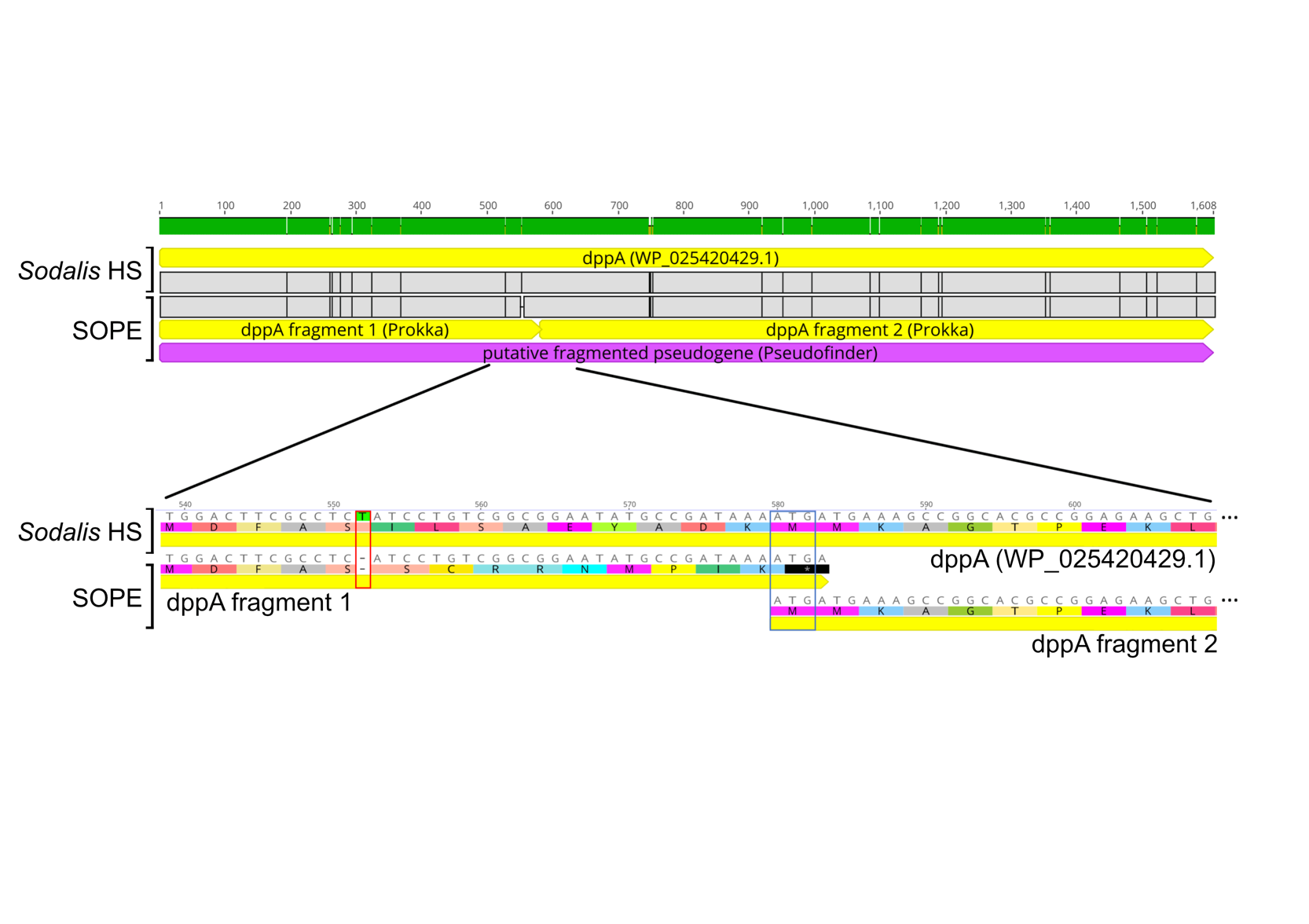
**

**Supplemental Figure S2** | Schematic showing an alignment between a reference gene and two query genes that have been fragmented. In this example, the *dppA* gene of *Ca.* Sodalis pierantonius str. SOPE, is compared to its counterpart in the reference genome *Sodalis* praecaptivus HS. This gene in SOPE was predicted as two different fragments (each with their own open reading frames), both of which align to the N- or C-termini of the reference gene. Pseudofinder will detect these kinds of events and, as part of the output, provide the user with a full, reconstructed gene sequence (shown in purple). The bottom inset shows the nucleotide sequence and corresponding translations, demonstrating how a single-nucleotide deletion (red rectangle at position 552) shifts the frame, resulting in an early, truncating stop codon in position 581, followed by the second predicted fragment of the *dppA* gene.

**
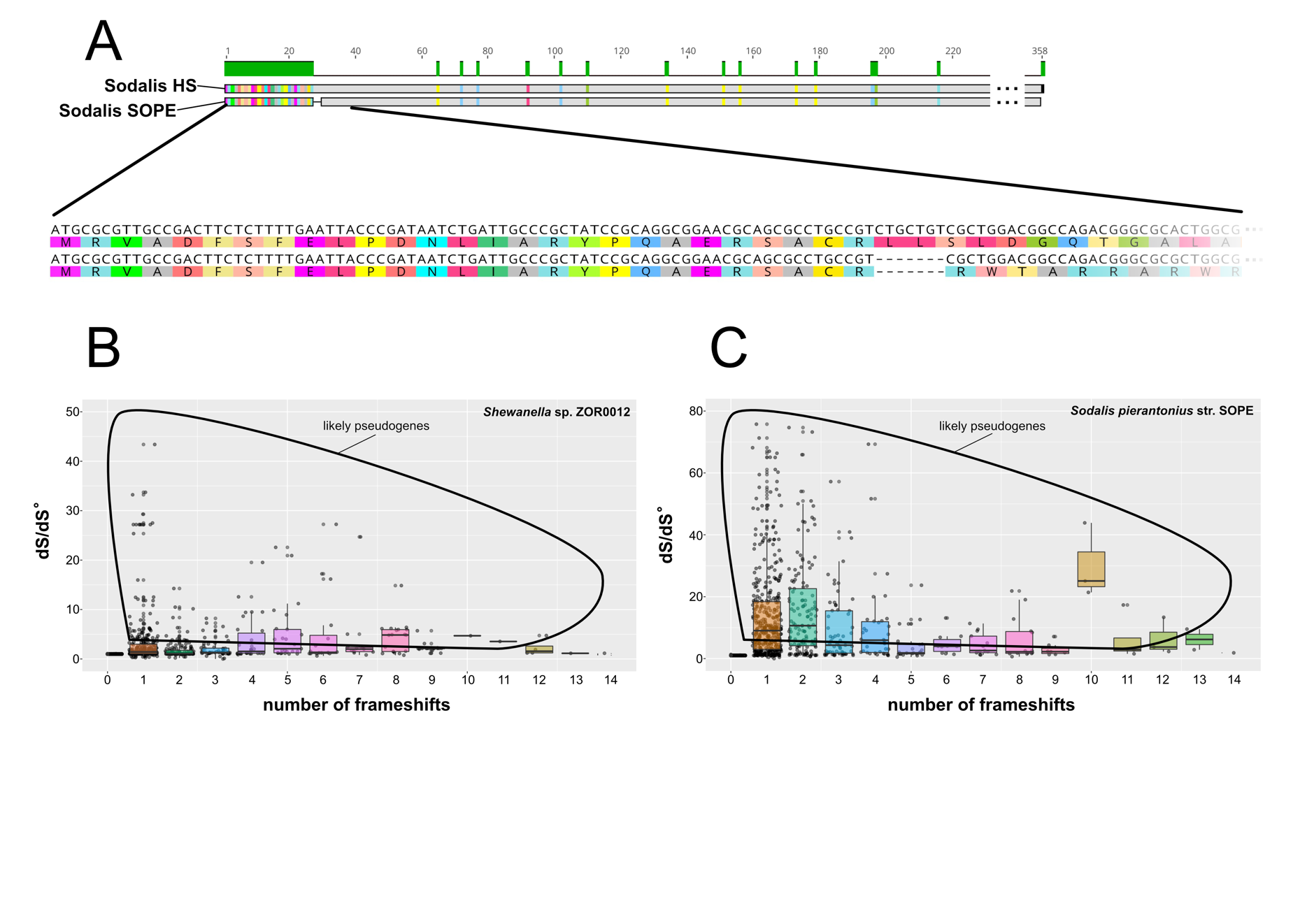
Supplemental Figure S3** | A) Schematic demonstrating how a 7-bp deletion in *Ca.* Sodalis pierantonius str. SOPE’s *queA* gene (bottom) results in an incorrect translation and a poor downstream alignment. The bottom inset shows the corresponding nucleotide and peptide alignments. These two alignments will result in two different estimates of evolutionary divergence, measured as *dS* (the rate of synonymous substitutions). Pseudofinder’s *Sleuth* module measures the difference between these two *dS* values, one calculated from the protein-dependent codon alignment (*dS*, shown in the top alignment), and one calculated directly from the nucleotide alignment (*dS˚*, bottom alignment). Thus, the *dS/dS˚* metric allows for a way to measure the extent to which frameshift-inducing indels impact the final protein sequence. Panels B and C show the number of frameshift-inducing indels (per-gene), plotted against the impact (*dS/dS˚*) of those frameshifts on the overall reading frame of the protein, in B) *Shewanella* sp. ZOR0012 and C) *Ca*. Sodalis pierantonius str. SOPE.
